# Correlation between the white blood cell/platelet ratio and 28-day all-cause mortality in cardiac arrest patients: a retrospective cohort study based on machine learning

**DOI:** 10.3389/fphar.2024.1527664

**Published:** 2025-01-07

**Authors:** Huai Huang, Guangqin Ren, Shanghui Sun, Zhi Li, Yongtian Zheng, Lijuan Dong, Shaoliang Zhu, Xiaosheng Zhu, Wenyu Jiang

**Affiliations:** ^1^ Department of Neurological Rehabilitation, Jiangbin Hospital of Guangxi Zhuang Autonomous Region, Nanning, China; ^2^ Department of Anesthesiology, Zhongshan Hospital of Traditional Chinese Medicine Affiliated to Guangzhou University of Traditional Chinese Medicine, Zhongshan, China; ^3^ Department of Nursing, Zhongshan Hospital of Traditional Chinese Medicine Affiliated to Guangzhou University of Traditional Chinese Medicine, Zhongshan, China; ^4^ Department of Endocrinology, Zhongshan Hospital of Traditional Chinese Medicine Affiliated to Guangzhou University of Traditional Chinese Medicine, Zhongshan, China; ^5^ Department of Hepatobiliary, Pancreas and Spleen Surgery, The People’s Hospital of Guangxi Zhuang Autonomous Region, Guangxi Academy of Medical Sciences, Nanning, China; ^6^ Department of Radiation, Jiangbin Hospital of Guangxi Zhuang Autonomous Region, Nanning, China

**Keywords:** cardiac arrest (CA), cardiopulmonary resuscitation (CPR), white blood cell to platelet ratio (WPR), 8-day all-cause mortality, prognosis

## Abstract

**Objective:**

This study aims to evaluate the association between the white blood cell-to-platelet ratio (WPR) and 28-day all-cause mortality among patients experiencing cardiac arrest.

**Methods:**

Utilizing data from 748 cardiac arrest patients in the Medical Information Mart for Intensive Care-IV (MIMIC-IV) 2.2 database, machine learning algorithms, including the Boruta feature selection method, random forest modeling, and SHAP value analysis, were applied to identify significant prognostic biomarkers. Key patient characteristics, encompassing demographic data, comorbidities, hematological and biochemical indices, and vital signs, were extracted using PostgreSQL Administration Tool (pgAdmin) software. The Cox proportional hazards model assessed the impact of WPR on mortality outcomes, while Kaplan-Meier survival curves and restricted cubic spline (RCS) analysis further validated the findings. Subgroup analyses stratified the prognostic value of WPR by demographic and clinical factors.

**Results:**

WPR demonstrated the highest prognostic significance among the variables studied, showing a strong association with 28-day all-cause mortality. In the unadjusted Model 1, hazard ratios (HRs) for WPR quartiles ranged from 1.88 (95% CI: 1.22–2.90) in Q2 to 3.02 (95% CI: 2.04–4.47) in Q4 (Ptrend <0.05). Adjusted models (Models 2–4) confirmed the robustness of these associations, even after accounting for demographic and clinical covariates. Kaplan-Meier and RCS analyses revealed a significant U-shaped relationship between WPR and mortality risk. Subgroup analyses indicated that elevated WPR was particularly associated with increased mortality in males, elderly patients, married individuals, and those with chronic pulmonary disease.

**Conclusion:**

WPR serves as an independent and reliable prognostic biomarker for 28-day mortality in cardiac arrest patients. Its integration into clinical decision-making may enhance the early identification of high-risk patients and guide tailored therapeutic interventions.

## Introduction

Cardiac arrest (CA) is a critical medical emergency that poses a significant threat to human health and survival (Zhong et al., 2023). And CA is a significant global health issue, affecting over 300,000 individuals annually in the United States alone (Benjamin et al., 2019). Worldwide, the incidence of out-of-hospital cardiac arrest (OHCA) varies between 20 and 140 per 100,000 population annually, with survival rates often below 10% in many regions (Berdowski et al., 2010). In Europe, similar patterns have been observed, with an estimated 275,000 cases of OHCA reported annually (Gräsner et al., 2016). These figures underscore the need for effective intervention and prognostic markers to improve outcomes. This stark contrast highlights disparities in healthcare infrastructure, public awareness, and emergency response systems, emphasizing the urgent need for targeted research and tailored strategies in different regions.

The sudden loss of effective circulation and respiratory function can lead to immediate life-threatening situations, and timely intervention is crucial for improving patient outcomes. Despite advancements in emergency medical services and post-resuscitation care (Penketh and Nolan, 2022), the 28-day all-cause mortality rate among CA patients remains high (Li et al., 2023). This high mortality is largely attributable to post-cardiac arrest syndrome, a multifaceted condition involving ischemia-reperfusion injury, systemic inflammatory responses, and brain injury, which underscores the need for effective prognostic markers to guide clinical management (drie et al., 2002; Nolan et al., 2021). Identifying predictors of mortality and understanding the underlying biological mechanisms are essential for improving clinical management and patient prognosis.

The white blood cell/platelet ratio (WPR) is increasingly recognized as a valuable biomarker in various medical conditions, including cardiovascular diseases. WPR reflects the balance between pro-inflammatory and anti-inflammatory white blood cells, which is essential for maintaining immune homeostasis and responding to stressors like infections or tissue injury. In the context of CA, the immune response significantly influences disease severity and recovery outcomes, with elevated WPR often associated with poorer prognosis and higher mortality risk (Çiçek et al., 2016; Gasparyan et al., 2019; Mayne et al., 2022). Platelets, in addition to their role in hemostasis, have emerged as key mediators in inflammation through the release of cytokines and interaction with immune cells, making their inclusion in a biomarker ratio such as WPR highly relevant in critical illnesses (Koupenova et al., 2018).

Previous studies have indicated that changes in white blood cell ratios, such as the neutrophil-to-lymphocyte ratio (NLR) and the monocyte-to-lymphocyte ratio (MLR), are associated with the severity of various inflammatory and cardiovascular diseases and predict poor outcomes (Mayne et al., 2022; Phan et al., 2020; Bg et al., 2021; Suner and Carr, 2020; Wang et al., 2023). However, the relationship between the WPR and 28-day all-cause mortality in CA patients has not been extensively studied. And these ratios predominantly focus on the inflammatory axis without integrating hematological parameters like platelet counts, which also reflect the coagulative and inflammatory states critical to CA pathophysiology. WPR has the potential to bridge this gap by combining inflammatory and coagulative biomarkers into a single prognostic parameter, yet its utility in CA has not been systematically evaluated.

With the advent of machine learning, we now have the capability to analyze complex datasets and identify patterns that may not be apparent through traditional statistical methods (Ogunjobi et al., 2024; Kang et al., 2022; Feng et al., 2023). Machine learning algorithms can handle large volumes of clinical data (Chattopadhyay, 2024) to uncover subtle relationships between variables, such as white blood cell ratios and patient outcomes after CA. Unlike traditional multivariable regression, machine learning can model nonlinear interactions and account for the multifactorial nature of critical illnesses, offering a more nuanced understanding of prognostic markers like WPR (Rajkomar et al., 2019). This advantage is particularly relevant in CA, where heterogeneous patient profiles and multifaceted pathophysiology complicate risk stratification.

CA patients often experience a state of stress due to abnormal activation of the immune system and the massive release of various cytokines. However, it remains unclear whether the WPR is associated with poor outcomes in critically ill CA patients (Cunningham et al., 2022). Furthermore, existing studies on prognostic markers in CA are often limited by small sample sizes, lack of external validation, and reliance on traditional statistical approaches. This study addresses these gaps by employing machine learning techniques on a large, well-curated dataset to explore the prognostic value of WPR in predicting 28-day all-cause mortality.

The WPR was selected as the biomarker of interest in this study due to its unique ability to integrate inflammatory and coagulative parameters, both of which play central roles in the pathophysiology of CA. CA triggers systemic responses such as ischemia-reperfusion injury and inflammatory cytokine surges, where white blood cells mediate immune responses and platelets, beyond their hemostatic role, actively participate in inflammation through cytokine release and immune cell interaction. WPR captures this interplay, offering a holistic measure of the inflammatory-coagulative balance, which is critical for assessing outcomes in critically ill patients (Zhang et al., 2023). Furthermore, previous studies have highlighted the prognostic value of hematological ratios like the neutrophil-to-lymphocyte ratio (NLR) and platelet-to-lymphocyte ratio (PLR) in cardiovascular and inflammatory diseases, underscoring the utility of such biomarkers in reflecting systemic physiological changes (Tudurachi et al., 2023). Unlike these markers, WPR bridges a gap in existing research by addressing both inflammation and coagulation. This makes WPR a robust and comprehensive biomarker for evaluating the prognosis of CA patients.

Therefore, this study aims to evaluate the relationship between the WPR and adverse outcomes in critically ill CA patients. By employing machine learning techniques to retrospectively analyze a cohort of CA patients, we aim to investigate the correlation between white blood cell ratios and 28-day all-cause mortality. Through this approach, we hope to identify potential prognostic indicators that can help in the early identification of high-risk patients and guide more personalized treatment strategies. By integrating WPR into a machine learning framework, this study seeks to contribute novel insights into the interplay between inflammation, coagulation, and survival outcomes, ultimately informing future research and clinical decision-making.

## Methods

### Data source

This study used data from MIMIC-IV 2.2, an electronic health record dataset comprising over 50,000 patients admitted to the intensive care units (ICUs) at Beth Israel Deaconess Medical Center (BIDMC) in Boston, Massachusetts, from 2008 to 2019. The Institutional Review Board of BIDMC approved the waiver of informed consent and the sharing of research resources.

The sample size for this study was determined based on previous research and statistical power analysis. To ensure adequate power to detect significant differences in 28-day mortality rates, we used a conservative effect size based on the expected differences in white blood cell/platelet ratio (WPR) between groups with different mortality outcomes. A power of 80% and a significance level of 0.05 were chosen, which is standard for clinical studies of this nature.

### Inclusion and exclusion criteria

The inclusion and exclusion criteria are shown in Table 1. Exclusion of Patients with No ACLS Treatment: The exclusion of patients who did not receive Advanced Cardiovascular Life Support (ACLS) treatment is critical for the integrity of the study. ACLS is a standardized, evidence-based approach to managing CA that includes advanced airway management, medication administration, and defibrillation. Patients who do not receive ACLS treatment may not undergo the same intensive resuscitation efforts, potentially leading to different outcomes that are not comparable to those in the study population. Excluding these patients ensures that the study specifically focuses on individuals who received consistent, high-standard care, thereby minimizing variability in treatment and improving the reliability of our results. This exclusion criterion allows us to more accurately assess the impact of inflammatory biomarkers like WPR in patients who underwent full resuscitation efforts, which is a critical aspect of the study’s design and objective.

**TABLE 1:** Inclusion and exclusion criteria.

Criteria type	Description
Inclusion criteria	
Age	≥18 years
Diagnosis	Medical records confirming cardiac arrest
Time Window	Patients who experienced a cardiac arrest event within the past 12 months
Exclusion Criteria	
Age	<18 years
Incomplete Data	Patients missing baseline WPR or other key clinical parameters
Non-Cardiac Arrest	Patients without confirmed cardiac arrest events
No ACLS Treatment	Patients who did not receive advanced cardiovascular life support (ACLS) treatment
Outside Time Window	Patients who experienced cardiac arrest events outside the 12-month time window

Outcomes:

Primary Outcome: 28-day all-cause mortality.

Secondary Outcome: In-hospital mortality.

### Data analysis

#### Data extraction

Data extraction was performed using pgAdmin software. Patient characteristics collected included age, gender, insurance, marital status, race, hospital stay duration, wbc, basophils_abs, eosinophils_abs, lymphocytes_abs, monocytes_abs, neutrophils_abs, basophils, eosinophils, lymphocytes, monocytes, neutrophils, hematocrit, hemoglobin, mch, mchc, mcv, platelet, rbc, rdw, scr_baseline, myocardial_infarct, congestive_heart_failure, peripheral_vascular_disease, cerebrovascular_disease, dementia, chronic_pulmonary_disease, rheumatic_disease, peptic_ulcer_disease, mild_liver_disease, diabetes_without_cc, diabetes_with_cc, paraplegia, renal_disease, malignant_cancer, severe_liver_disease, metastatic_solid_tumor, aids, albumin, aniongap, bicarbonate, bun, calcium, chloride, creatinine, glucose, sodium, potassium, crp, alt, alp, ast, amylase, bilirubin_total, bilirubin_direct, bilirubin_indirect, ck_cpk, ck_mb, ld_ldh, lactate, apsiii, heart_rate, sbp, dbp, spo2, urineoutput_24 h, neurologic, cardiovascular, renal, pulmonary, gcs, urineoutput, hourly_patient_fluid_removal, ventilation_duration. Relevant blood combination index calculation formulas are as follows: NLR = neutrophils/lymphocytes, LMR = lymphocytes/monocytes, PLR = platelet/lymphocytes, NMR = neutrophils/monocytes, WPR = wbc/platelet, MCV = hematocrit/rbc, MCH = hemoglobin/rbc, MCHC = hemoglobin/hematocrit, RDW_CV = rdw/MCV, HHR = hemoglobin/hematocrit, HBR = hemoglobin/rbc.

#### Cox model with Boruta, RF, and SHAP analysis

We used the Cox proportional hazards model to assess the impact of each feature on survival time. During feature selection, the Boruta algorithm, based on 500 random forest trees, was employed to identify the most relevant features for survival analysis. The Boruta algorithm outputs features as confirmed, tentative, or rejected, guiding subsequent model construction and interpretation. Additionally, the Random Survival Forest (RSF) method was used to explore factors influencing patient survival. Data were retrieved from MIMIC-IV 2.2, including patient hospital stay (time to event or last follow-up time) and event status (survival or death). Analysis was performed using the randomForestSRC package in R. A random forest survival model (rfsrc function) was constructed with survival time and event status as primary endpoints. Predictive variables included Neutrophil-to-Lymphocyte Ratio (NLR), Lymphocyte-to-Monocyte Ratio (LMR), Platelet-to-Lymphocyte Ratio (PLR), Neutrophil-to-Monocyte Ratio (NMR), WPR, Mean Corpuscular Volume (MCV), Mean Corpuscular Hemoglobin (MCH), Mean Corpuscular Hemoglobin Concentration (MCHC), Red Cell Distribution Width-Coefficient of Variation (RDW_CV), Heart rate reserve (HHR), and High Bleeding Risk (HBR). Cross-validation and resampling methods were used to avoid overfitting and validate model predictive performance. SHAP values were introduced to understand feature impact mechanisms, calculated using the treeshap package, revealing each feature’s contribution to individual predictions. SHAP value visualization helped interpret model predictions and feature importance, uncovering specific impact pathways of different features on patient prognosis.

#### Statistical analysis

Since this study is a retrospective analysis, no sample size calculation was performed. Variables with more than 20% missing data were excluded. For variables with less than 20% missing data, the random forest algorithm was used to impute missing values. Random forest, a robust machine learning method, constructs multiple decision trees and aggregates their predictions to handle missing information. Patients were divided into four groups based on WPR quartiles. Normally distributed continuous variables were expressed as mean (standard deviation [SD]) and analyzed using analysis of variance (ANOVA). Non-normally distributed variables were analyzed using the Mann-Whitney *U* test or Kruskal–Wallis test. Categorical variables were expressed as numbers and percentages and analyzed using the χ2 test or Fisher’s exact test. Kaplan-Meier survival curves and log-rank tests were used to compare in-hospital and 28-day survival rates across WPR groups. Proportional hazards regression models (Cox regression models) were used to assess hazard ratios (HR) and 95% confidence intervals (95% CI). Model 1 was unadjusted; Model 2 adjusted for age, gender, insurance, marital status, heart rate, sbp, dbp, and spo2. Model 3 further adjusted for age, gender, insurance, marital status, wbc, basophils_abs, eosinophils_abs, lymphocytes_abs, monocytes_abs, neutrophils_abs, basophils, eosinophils, lymphocytes, monocytes, neutrophils, hematocrit, hemoglobin, mch, mchc, mcv, platelet, rbc, rdw, albumin, aniongap, bicarbonate, bun, calcium, chloride, creatinine, glucose, sodium, potassium, crp, alt, alp, ast, amylase, bilirubin_total, bilirubin_direct, bilirubin_indirect, ck_cpk, ck_mb, ld_ldh, lactate, apsiii, heart_rate, sbp, dbp, spo2, urineoutput_24 h, and gcs. Model 4 included adjustments for age, gender, insurance, marital status, wbc, basophils_abs, eosinophils_abs, lymphocytes_abs, monocytes_abs, neutrophils_abs, basophils, eosinophils, lymphocytes, monocytes, neutrophils, hematocrit, hemoglobin, mch, mchc, mcv, platelet, rbc, rdw, peripheral_vascular_disease, cerebrovascular_disease, dementia, chronic_pulmonary_disease, rheumatic_disease, peptic_ulcer_disease, mild_liver_disease, diabetes_with_cc, paraplegia, renal_disease, malignant_cancer, severe_liver_disease, metastatic_solid_tumor, aids, albumin, aniongap, bicarbonate, bun, calcium, chloride, creatinine, glucose, sodium, potassium, crp, alt, alp, ast, amylase, bilirubin_total, bilirubin_direct, bilirubin_indirect, ck_cpk, ck_mb, ld_ldh, lactate, apsiii, heart_rate, sbp, dbp, spo2, urineoutput_24h. Two-tailed *p*-value <0.05 was considered statistically significant. Statistical analyses were performed using R software (version 4.3.1).

#### Restricted cubic spline curve

In this study, we collected survival data (outcome variable); WPR (continuous predictor variable); and age, gender, insurance, marital status, wbc, basophils_abs, eosinophils_abs, lymphocytes_abs, monocytes_abs, neutrophils_abs, basophils, eosinophils, lymphocytes, monocytes, neutrophils, hematocrit, hemoglobin, mch, mchc, mcv, platelet, rbc, rdw, peripheral_vascular_disease, cerebrovascular_disease, dementia, chronic_pulmonary_disease, rheumatic_disease, peptic_ulcer_disease, mild_liver_disease, diabetes_with_cc, paraplegia, renal_disease, malignant_cancer, severe_liver_disease, metastatic_solid_tumor, aids, albumin, aniongap, bicarbonate, bun, calcium, chloride, creatinine, glucose, sodium, potassium, crp, alt, alp, ast, amylase, bilirubin_total, bilirubin_direct, bilirubin_indirect, ck_cpk, ck_mb, ld_ldh, lactate, apsiii, heart_rate, sbp, dbp, spo2, urineoutput_24 h usage (covariates). Restricted cubic spline (RCS) Cox regression models were used to examine the potential nonlinear relationship between changes in WPR and survival. The number of knots ranged from 3 to 7, and the RCS model with the lowest Akaike Information Criterion (AIC) value was selected.

#### Subgroup analysis

Subgroup analysis was performed according to WPR quartiles. Patients were divided into two groups by age (<65 years, ≥65 years). Other subgroups included gender, insurance, marital status, peripheral_vascular_disease, cerebrovascular_disease, dementia, chronic_pulmonary_disease, rheumatic_disease, peptic_ulcer_disease, mild_liver_disease, diabetes, paraplegia, renal_disease, malignant_cancer, severe_liver_disease, metastatic_solid_tumor, aids. Cox proportional hazards regression analysis was conducted for each subgroup, reporting hazard ratios (HRs) and 95% confidence intervals (CIs).

In this study, we employed machine learning techniques to analyze the data and identify significant predictors of 28-day mortality. Initially, the dataset was pre-processed to handle missing values and outliers. The primary variables included inflammatory biomarkers (such as NLR, LMR, and WPR) and clinical parameters. A random forest survival model (using the rfsrc function in the randomForestSRC package in R) was built with survival time and event status (survival or death) as the primary endpoints. This method enabled us to evaluate the importance of various predictors in relation to patient survival.

### Machine learning process

To improve clarity, we have separated the machine learning methodology into a distinct section. The machine learning model was constructed using the randomForestSRC package in R, a powerful tool for analyzing survival data. The random forest survival model (rfsrc function) was used to predict the relationship between survival time and event status (survival or death). The features selected for the model included NLR, LMR, PLR, WPR, and other hematological parameters. The random forest algorithm was trained on the dataset, and its performance was assessed using cross-validation techniques to ensure robustness and generalizability. The importance of each variable was determined based on its contribution to the model’s predictive accuracy.

To evaluate the performance of the machine learning model, we employed k-fold cross-validation. This method divides the dataset into k equally sized subsets (or “folds”). The model is then trained on k-1 folds, while the remaining fold is used as a test set to evaluate the model’s performance. This process is repeated k times, with each fold serving as the test set once. The final performance metric is averaged over all folds to provide a more robust estimate of model accuracy, reducing the risk of overfitting and ensuring that the model generalizes well to unseen data. For this study, we used 5-fold cross-validation, meaning the dataset was divided into 5 subsets. In each iteration, 80% of the data was used for training, and 20% was used for testing. This approach helps mitigate the risk of selection bias and ensures that every observation is used for both training and testing.

### Handling of potential confounding factors

In this study, we carefully considered potential confounding factors, such as comorbidities and medications, which could influence the relationship between hematological markers (e.g., WPR, NLR, PLR) and patient outcomes. To minimize bias and ensure the robustness of our findings, we applied the following strategies:

1. Comorbidities: Key comorbidities, such as hypertension, diabetes, coronary artery disease, and chronic kidney disease, were recorded for all participants. These conditions are known to significantly influence both hematological markers and patient survival outcomes. We controlled for these variables in our analysis by including them as covariates in the multivariable regression models. This approach allows us to isolate the effect of the hematological markers on outcomes while accounting for the potential influence of these comorbidities.
2. Medications: Medications administered during hospitalization, including anticoagulants, antihypertensive drugs, and diabetic treatments, can also affect both hematological profiles and patient outcomes. To address this, we carefully documented all medications taken by the participants during their hospital stay. We included medication use as a covariate in our statistical models to control for any confounding effects these treatments may have had on the relationship between hematological markers and 28-day mortality.
3. Statistical Adjustment: In addition to including comorbidities and medications in our multivariable models, we used propensity score matching (PSM) to further balance confounders between different patient groups. PSM helps reduce bias by matching patients with similar characteristics (e.g., age, gender, comorbidities) but different outcomes, allowing for a more accurate estimate of the relationship between WPR and mortality.

By accounting for these confounding factors, we aimed to ensure that the observed relationships between hematological markers and outcomes were not driven by underlying medical conditions or treatments.

### Factors influencing 28-day mortality

Several factors contribute to the occurrence of 28-day mortality in patients after CA. In addition to hematological markers like the white blood cell-to-platelet ratio (WPR), the following factors have been consistently associated with increased mortality risk:

1. Age: Older age is a well-established risk factor for poor outcomes in CA patients. Studies have shown that elderly patients have higher mortality rates, often due to reduced physiological reserve and the presence of comorbidities like heart failure, diabetes, and renal dysfunction.
2. Initial Rhythm: The initial cardiac rhythm upon presentation is a key determinant of survival. Patients presenting with shockable rhythms such as ventricular fibrillation or pulseless ventricular tachycardia tend to have better survival rates compared to those with non-shockable rhythms (e.g., asystole or pulseless electrical activity).
3. Comorbidities: Pre-existing conditions such as coronary artery disease, hypertension, diabetes, and chronic kidney disease significantly influence mortality rates. These comorbidities often exacerbate the systemic response to CA, making recovery more difficult and increasing the risk of multi-organ failure.
4. Neurological Status: The neurological outcome, assessed by tools such as the Glasgow Coma Scale (GCS) upon hospital admission, is a crucial predictor of 28-day mortality. Severe neurological impairment is associated with poor long-term survival, as brain injury often leads to systemic complications and worse overall recovery.
5. Time to Resuscitation: The time from collapse to the initiation of cardiopulmonary resuscitation (CPR) is another critical factor. Faster initiation of CPR and defibrillation significantly improves survival rates, while prolonged time to treatment increases the likelihood of adverse outcomes, including irreversible brain damage.
6. Acidosis and Hypoxia: Metabolic acidosis and hypoxia during and after resuscitation are associated with increased mortality. Correcting acid-base imbalances and ensuring adequate oxygenation are essential for improving survival and recovery.

## Results

### COX + Boruta + RF + SHAP screening of prognostic hematological parameters

**FIGURE 1:**
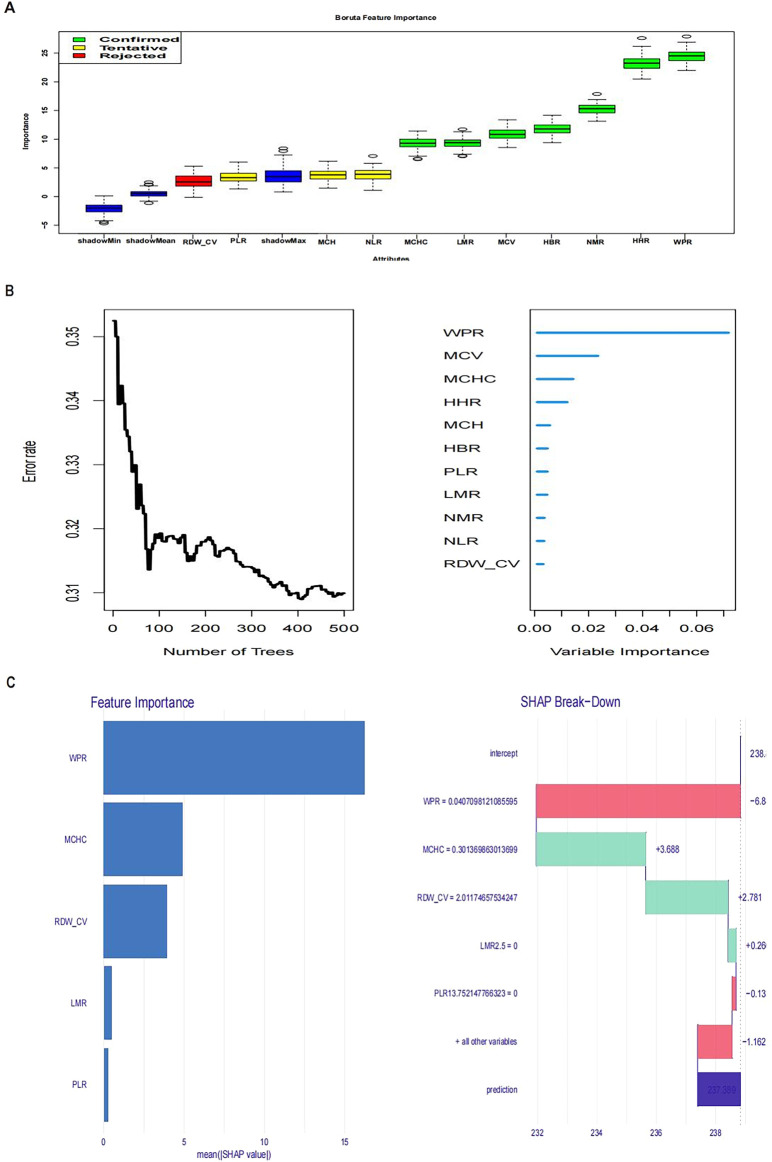
Figure **(A)** Boruta feature importance; Figure **(B)** Variable importance from random forest analysis; Figure **(C)**: SHAP values.

In this study, our primary objective was to evaluate the prognostic implications of various hematological markers in patients who experienced CA. Using advanced methodologies such as the Boruta algorithm, random forest modeling, and SHAP (Shapley Additive Explanations) value analysis, we assessed the influence of multiple hematological parameters on patient outcomes, as shown in the figure. The Boruta algorithm identified several key features, including the WPR, mean corpuscular hemoglobin concentration (MCHC), red cell distribution width-coefficient of variation (RDW-CV), and lymphocyte to monocyte ratio (LMR). These markers appear to be strongly associated with the inflammatory response and immune status of the patient, and they may serve as critical biomarkers for adverse outcomes in CA cases (Figure 1A). In the random forest model, WPR, MCHC, mean corpuscular volume (MCV), and high hemoglobin ratio (HHR) emerged as variables with high predictive importance. Of these, WPR exhibited the greatest prognostic significance, highlighting its potential role in reflecting both immune function and the risk of infection (Figure 1B). SHAP analysis further elucidated the contribution of individual features to the model’s predictions. While high WPR values were positively correlated with worse prognoses, lower MCHC values also indicated a poor clinical outcome. This analysis provided a detailed breakdown of how each feature influenced the overall predictive model, underscoring WPR as a particularly relevant marker for prognosis in CA (Figure 1C).

Together, these findings demonstrate that hematological parameters, particularly WPR, play a significant role in predicting survival outcomes in CA patients. The values of these biomarkers are closely tied to survival probabilities, offering vital insights for clinical decision-making. To further clarify the prognostic value of WPR, additional analyses were performed.

### Baseline characteristics and differential analysis of WPR

Data from 748 patients diagnosed with CA were extracted from the MIMIC-IV database and categorized into four quartiles based on the WPR (Group 1: 187 patients, Group 2: 186 patients, Group 3: 188 patients, Group 4: 187 patients). As shown in Table 2, no statistically significant differences were observed in age (mean 65.88 years ±16.44, F = 1.69, *p* = 0.167) or length of hospital stay (8.22–10.22 days, F = 1.53, *p* = 0.204). However, the overall mean white blood cell count was 11.32 ± 6.14, with Group 4 showing significantly higher values (F = 161.27, *p* < 0.001). Significant intergroup differences were also noted in absolute eosinophil count (F = 6.75, *p* < 0.001), absolute lymphocyte count (F = 9.43, *p* < 0.001), and both absolute and relative neutrophil counts (F = 68.04, F = 19.76, respectively, both *p* < 0.001). Hematocrit levels did not differ significantly among the groups (F = 1.11, *p* = 0.346), while hemoglobin levels approached significance (F = 2.57, *p* = 0.054). Platelet counts varied significantly, with the highest mean observed in Group 1 (203.96 ± 93.10, F = 45.87, *p* < 0.001).

**TABLE 2:** Baseline characteristics and differential analysis of WPR.

Variables	Total (n = 748)	Q1 (n = 187)	Q2 (n = 186)	Q3 (n = 188)	Q4 (n = 187)	Statistic	*P*
Age, Mean ± SD	65.88 ± 16.44	64.78 ± 14.46	66.77 ± 15.42	67.60 ± 16.85	64.35 ± 18.64	F = 1.69	0.167
Hospital Stay Duration, Mean ± SD	9.18 ± 11.90	9.99 ± 11.92	8.22 ± 12.91	8.28 ± 7.88	10.22 ± 13.97	F = 1.53	0.204
Wbc, Mean ± SD	11.32 ± 6.14	7.09 ± 3.00	9.23 ± 3.31	11.52 ± 4.18	17.41 ± 7.37	F = 161.27	<0.001
Basophils Abs, Mean ± SD	0.03 ± 0.02	0.03 ± 0.02	0.04 ± 0.02	0.03 ± 0.02	0.03 ± 0.02	F = 4.64	0.003
Eosinophils Abs, Mean ± SD	0.18 ± 0.18	0.23 ± 0.23	0.18 ± 0.13	0.15 ± 0.13	0.17 ± 0.20	F = 6.75	<0.001
Lymphocytes Abs, Mean ± SD	1.44 ± 0.59	1.40 ± 0.51	1.36 ± 0.38	1.38 ± 0.57	1.64 ± 0.77	F = 9.43	<0.001
Monocytes Abs, Mean ± SD	0.73 ± 0.27	0.68 ± 0.22	0.76 ± 0.20	0.72 ± 0.27	0.75 ± 0.35	F = 3.53	0.015
Neutrophils Abs, Mean ± SD	8.84 ± 4.38	6.65 ± 3.23	7.40 ± 2.30	9.41 ± 4.25	11.89 ± 5.16	F = 68.04	<0.001
Basophils, Mean ± SD	0.35 ± 0.34	0.44 ± 0.59	0.39 ± 0.17	0.31 ± 0.19	0.26 ± 0.19	F = 11.34	<0.001
Eosinophils, Mean ± SD	1.93 ± 1.79	2.79 ± 2.54	2.00 ± 1.18	1.57 ± 1.33	1.37 ± 1.44	F = 25.40	<0.001
Lymphocytes, Mean ± SD	14.46 ± 6.10	17.44 ± 6.30	14.62 ± 4.00	13.40 ± 7.09	12.36 ± 5.34	F = 26.76	<0.001
Monocytes, Mean ± SD	7.16 ± 2.54	8.02 ± 2.51	8.04 ± 1.96	6.72 ± 2.40	5.85 ± 2.56	F = 37.96	<0.001
Neutrophils, Mean ± SD	74.15 ± 8.65	70.36 ± 8.99	74.04 ± 5.54	75.90 ± 9.42	76.29 ± 8.80	F = 19.76	<0.001
Hematocrit, Mean ± SD	33.45 ± 6.59	32.75 ± 5.92	33.42 ± 5.96	33.92 ± 6.98	33.70 ± 7.37	F = 1.11	0.346
Hemoglobin, Mean ± SD	10.83 ± 2.30	10.45 ± 2.10	10.82 ± 2.08	11.03 ± 2.36	11.00 ± 2.59	F = 2.57	0.054
Platelet, Mean ± SD	203.96 ± 93.10	258.01 ± 120.34	211.34 ± 75.04	189.83 ± 66.36	156.75 ± 69.79	F = 45.87	<0.001
Rbc, Mean ± SD	3.66 ± 0.77	3.63 ± 0.73	3.67 ± 0.69	3.70 ± 0.79	3.63 ± 0.84	F = 0.37	0.778
Rdw, Mean ± SD	15.28 ± 2.05	15.51 ± 2.31	15.20 ± 2.00	15.22 ± 2.02	15.19 ± 1.84	F = 1.00	0.390
Scr Baseline, Mean ± SD	1.28 ± 1.23	1.44 ± 1.68	1.22 ± 0.90	1.28 ± 1.19	1.17 ± 0.98	F = 1.68	0.169
Albumin, Mean ± SD	3.03 ± 0.44	3.14 ± 0.46	3.13 ± 0.38	3.00 ± 0.41	2.86 ± 0.45	F = 17.52	<0.001
Aniongap, Mean ± SD	16.02 ± 4.99	14.64 ± 3.76	15.12 ± 4.52	16.05 ± 4.61	18.27 ± 6.02	F = 21.04	<0.001
Bicarbonate, Mean ± SD	23.04 ± 4.99	25.03 ± 4.70	24.15 ± 4.32	22.57 ± 4.95	20.39 ± 4.71	F = 35.42	<0.001
Bun, Mean ± SD	32.97 ± 22.70	29.62 ± 19.89	31.51 ± 19.45	34.52 ± 24.41	36.23 ± 25.91	F = 3.23	0.022
Calcium, Mean ± SD	8.62 ± 0.95	8.80 ± 0.74	8.77 ± 0.88	8.46 ± 0.98	8.46 ± 1.12	F = 7.45	<0.001
Chloride, Mean ± SD	102.90 ± 6.20	101.96 ± 5.58	102.40 ± 6.17	103.46 ± 6.36	103.78 ± 6.54	F = 3.61	0.013
Creatinine, Mean ± SD	1.95 ± 1.88	1.97 ± 2.31	1.72 ± 1.53	1.97 ± 1.88	2.16 ± 1.71	F = 1.69	0.168
Glucose, Mean ± SD	154.03 ± 70.20	131.86 ± 59.82	141.94 ± 50.43	159.25 ± 63.53	182.98 ± 90.09	F = 20.50	<0.001
Sodium, Mean ± SD	138.94 ± 4.79	139.06 ± 4.42	138.83 ± 4.80	138.81 ± 4.62	139.06 ± 5.32	F = 0.15	0.927
Potassium, Mean ± SD	4.36 ± 0.73	4.25 ± 0.71	4.29 ± 0.62	4.38 ± 0.66	4.51 ± 0.89	F = 4.82	0.002
Crp, Mean ± SD	92.98 ± 35.40	77.72 ± 34.81	89.58 ± 32.03	99.44 ± 36.95	105.13 ± 31.51	F = 23.54	<0.001
Alt, Mean ± SD	213.58 ± 591.15	130.63 ± 414.58	118.35 ± 216.79	215.29 ± 575.74	389.54 ± 896.93	F = 8.62	<0.001
Alp, Mean ± SD	113.44 ± 79.69	114.07 ± 74.42	117.21 ± 90.77	111.93 ± 80.57	110.59 ± 72.24	F = 0.24	0.866
Ast, Mean ± SD	335.95 ± 950.45	196.53 ± 785.03	157.99 ± 415.36	314.18 ± 876.64	674.27 ± 1,378.66	F = 11.95	<0.001
Amylase, Mean ± SD	115.57 ± 67.45	113.66 ± 41.20	97.56 ± 57.11	110.74 ± 81.01	140.25 ± 75.96	F = 13.81	<0.001
Bilirubin Total, Mean ± SD	0.90 ± 0.93	0.79 ± 0.79	0.81 ± 0.67	0.86 ± 0.73	1.15 ± 1.33	F = 6.42	<0.001
Bilirubin Direct, Mean ± SD	1.75 ± 0.98	1.77 ± 0.85	1.61 ± 0.94	1.73 ± 1.18	1.88 ± 0.89	F = 2.51	0.058
Bilirubin Indirect, Mean ± SD	0.96 ± 0.41	0.93 ± 0.31	0.86 ± 0.35	0.93 ± 0.40	1.12 ± 0.49	F = 15.72	<0.001
Ck Cpk, Mean ± SD	1,353.44 ± 7,765.71	733.26 ± 2022.74	631.94 ± 1,092.47	1,117.14 ± 1733.19	2,928.81 ± 15,180.25	F = 3.59	0.013
Ck Mb, Mean ± SD	30.44 ± 56.01	13.99 ± 15.95	18.74 ± 23.72	33.61 ± 55.71	55.35 ± 87.31	F = 22.38	<0.001
Ld Ldh, Mean ± SD	679.85 ± 1,014.33	517.75 ± 932.11	462.39 ± 673.73	641.87 ± 785.21	1,096.45 ± 1,393.62	F = 15.95	<0.001
Lactate, Mean ± SD	3.50 ± 2.94	2.84 ± 2.31	2.87 ± 2.05	3.24 ± 2.57	5.04 ± 3.90	F = 25.82	<0.001
Apsiii, Mean ± SD	64.05 ± 27.92	58.00 ± 24.41	57.39 ± 22.48	65.22 ± 28.49	75.54 ± 31.64	F = 18.29	<0.001
Heart Rate, Mean ± SD	88.03 ± 14.66	84.34 ± 12.02	84.17 ± 10.97	90.04 ± 16.18	93.52 ± 16.53	F = 19.52	<0.001
Sbp, Mean ± SD	121.82 ± 18.60	118.64 ± 14.59	124.55 ± 20.15	124.61 ± 17.68	119.49 ± 20.68	F = 5.65	<0.001
Mbp, Mean ± SD	82.43 ± 14.35	78.63 ± 11.02	84.65 ± 15.54	84.97 ± 12.96	81.46 ± 16.41	F = 8.35	<0.001
Temperature, Mean ± SD	36.36 ± 0.79	36.57 ± 0.58	36.48 ± 0.58	36.33 ± 0.93	36.06 ± 0.92	F = 15.48	<0.001
Spo2, Mean ± SD	95.56 ± 5.17	96.59 ± 2.64	96.71 ± 3.23	95.57 ± 5.27	93.37 ± 7.42	F = 17.89	<0.001
Urineoutput 24 h, Mean ± SD	236.13 ± 227.58	213.45 ± 156.77	245.28 ± 327.23	244.30 ± 200.47	241.48 ± 189.27	F = 0.83	0.476
Neurologic, Mean ± SD	1.23 ± 1.70	1.09 ± 1.53	0.92 ± 1.37	1.27 ± 1.75	1.65 ± 2.02	F = 6.46	<0.001
Cardiovascular, Mean ± SD	1.14 ± 1.18	0.85 ± 0.87	1.12 ± 1.10	1.22 ± 1.23	1.38 ± 1.40	F = 6.69	<0.001
Renal, Mean ± SD	3.01 ± 1.51	2.81 ± 1.40	2.85 ± 1.42	2.98 ± 1.58	3.41 ± 1.57	F = 6.30	<0.001
Pulmonary, Mean ± SD	1.38 ± 1.20	1.09 ± 1.07	1.15 ± 1.07	1.50 ± 1.20	1.78 ± 1.30	F = 14.20	<0.001
Gcs, Mean ± SD	14.31 ± 1.69	14.20 ± 1.76	14.36 ± 1.73	14.55 ± 1.06	14.11 ± 2.03	F = 2.53	0.056
Urineoutput, Mean ± SD	240.44 ± 233.93	221.69 ± 180.52	251.70 ± 328.97	250.09 ± 204.78	238.30 ± 191.85	F = 0.65	0.580
Hourly Patient Fluid Removal, Mean ± SD	146.75 ± 71.61	129.12 ± 57.55	139.77 ± 57.63	159.60 ± 81.56	158.42 ± 81.41	F = 8.28	<0.001
Ventilation Duration, Mean ± SD	36.13 ± 40.22	32.05 ± 31.42	29.73 ± 34.57	40.19 ± 44.41	42.47 ± 47.16	F = 4.46	0.004

F, ANOVA.

SD, standard deviation.

Significant differences were also identified in key biochemical markers, including the anion gap (F = 21.04, *p* < 0.001), bicarbonate (F = 35.42, *p* < 0.001), blood urea nitrogen (F = 3.23, *p* = 0.022), calcium (F = 7.45, *p* < 0.001), chloride (F = 3.61, *p* = 0.013), glucose (F = 20.50, *p* < 0.001), potassium (F = 4.82, *p* = 0.002), C-reactive protein (F = 23.54, *p* < 0.001), and liver enzymes (ALT: F = 8.62, *p* < 0.001; AST: F = 11.95, *p* < 0.001), as well as amylase (F = 13.81, *p* < 0.001) and lactate (F = 25.82, *p* < 0.001). Group 4, in particular, exhibited more severe deviations across several physiological and biochemical parameters, indicating an elevated health risk in these patients. The results of the ANOVA F-tests and associated *p*-values reveal significant intergroup differences in various key physiological and biochemical parameters. These variations likely reflect underlying disparities in health status, treatment outcomes, or demographic characteristics among the patient groups. The findings provide important statistical evidence to guide clinical decision-making and inform future research.

### Baseline characteristics and differential analysis of relevant hematological parameters

This report provides a detailed analysis of the hematological indicators of 748 participants, revealing significant statistical differences among the groups, highlighting potential health conditions and pathological changes (Table 3). The neutrophil-to-lymphocyte ratio (NLR) data showed that the average value in Group 4 was 8.33 ± 7.94, significantly higher than the 5.84 ± 4.28 observed in Groups 1, 2, and 3 (F = 4.57, *p* = 0.004), indicating a possibly more severe inflammatory state in Group 4. The average lymphocyte-to-monocyte ratio (LMR) in Group 4 was 2.61 ± 2.27, compared to 1.92 ± 0.79 in Group 2 (F = 4.90, *p* = 0.002), reflecting different levels of immune activation and risk of thrombosis or inflammatory response. Additionally, this suggests a higher risk of infection.The mean corpuscular volume (MCV), mean corpuscular hemoglobin (MCH), and mean corpuscular hemoglobin concentration (MCHC) in Group 4 were 9.34 ± 0.80, 3.04 ± 0.27, and 0.33 ± 0.02, respectively (F values were 4.64, 11.34, and 6.80, respectively, all *p* < 0.001). The red cell distribution width coefficient of variation (RDW CV) and high hemoglobin ratio (HHR) also showed significant differences (F = 3.03 and 6.80, *p* < 0.05), indicating potential hematological disorders and malnutrition.These detailed numerical data and significant results provide a solid foundation for clinical research and indicate important directions for future treatment strategies and health management.

**TABLE 3:** Baseline characteristics and differential analysis of hematological parameters.

Variables	Total (n = 748)	Q1 (n = 187)	Q2 (n = 186)	Q3 (n = 188)	Q4 (n = 187)	Statistic	*P*
NLR, Mean ± SD	7.10 ± 7.68	6.33 ± 10.32	5.84 ± 4.28	7.87 ± 6.70	8.33 ± 7.94	F = 4.57	0.004
LMR, Mean ± SD	2.29 ± 1.86	2.45 ± 2.34	1.92 ± 0.79	2.20 ± 1.53	2.61 ± 2.27	F = 4.90	0.002
PLR, Mean ± SD	19.27 ± 25.88	23.32 ± 42.14	16.54 ± 11.03	20.61 ± 23.26	16.57 ± 14.68	F = 3.09	0.027
NMR, Mean ± SD	12.68 ± 10.81	10.41 ± 9.09	10.45 ± 7.09	13.47 ± 8.63	16.36 ± 15.39	F = 13.63	<0.001
MCV, Mean ± SD	9.19 ± 0.69	9.08 ± 0.71	9.15 ± 0.56	9.20 ± 0.66	9.34 ± 0.80	F = 4.64	0.003
MCH, Mean ± SD	2.97 ± 0.25	2.89 ± 0.28	2.96 ± 0.21	2.99 ± 0.22	3.04 ± 0.27	F = 11.34	<0.001
MCHC, Mean ± SD	0.32 ± 0.02	0.32 ± 0.02	0.32 ± 0.02	0.33 ± 0.02	0.33 ± 0.02	F = 6.80	<0.001
RDW CV, Mean ± SD	1.67 ± 0.28	1.72 ± 0.34	1.67 ± 0.25	1.67 ± 0.26	1.64 ± 0.25	F = 3.03	0.029
HHR, Mean ± SD	0.32 ± 0.02	0.32 ± 0.02	0.32 ± 0.02	0.33 ± 0.02	0.33 ± 0.02	F = 6.80	<0.001
HBR, Mean ± SD	2.97 ± 0.25	2.89 ± 0.28	2.96 ± 0.21	2.99 ± 0.22	3.04 ± 0.27	F = 11.34	<0.001

F, ANOVA.

SD, standard deviation.

### 28-Day all-cause mortality and in-hospital mortality

Regarding 28-day mortality and in-hospital mortality, the mortality rate was higher in quartile 4 (Table 4). In the Cox regression analysis, using quartile 1 as the reference, the risk of death significantly increased in quartiles 3 and 4 (Table 5). The Kaplan-Meier curves illustrate the mortality risk. According to the Cox proportional hazards regression model, the inflection point was located at quartile 1, where the risk of death was minimal, thus demonstrating consistency between the two outcomes.

**TABLE 4:** 28-day all-cause mortality and in-hospital mortality.

Variables	Total (n = 748)	Q1 (n = 187)	Q2 (n = 186)	Q3 (n = 188)	Q4 (n = 187)	Statistic	*P*
28-day all-cause mortality, n(%)						χ^2^ = 48.75	<0.001
Alive	475 (63.50)	148 (79.14)	132 (70.97)	106 (56.38)	89 (47.59)		
Dead	273 (36.50)	39 (20.86)	54 (29.03)	82 (43.62)	98 (52.41)		
28 days, n (%)						χ^2^ = 54.42	<0.001
Alive	483 (64.57)	153 (81.82)	133 (71.51)	106 (56.38)	91 (48.66)		
Dead	265 (35.43)	34 (18.18)	53 (28.49)	82 (43.62)	96 (51.34)		

χ^2^, Chi-square test.

**TABLE 5:** Cox regression model (28-day all-cause mortality).

Variables	Model1		Model2		Model3		Model4	
	HR (95%CI)	Ptrend	HR (95%CI)	Ptrend	HR (95%CI)	P	HR (95%CI)	Ptrend
WPR quantile								
Q1	1.00 (Reference)		1.00 (Reference)		1.00 (Reference)		1.00 (Reference)	
Q2	1.64 (1.09–2.48)	0.019	1.54 (1.01–2.36)	0.045	1.79 (1.06–3.01)	0.028	1.79 (1.06–3.01)	0.028
Q3	2.29 (1.56–3.35)	<0.001	2.02 (1.36–3.02)	<0.001	2.82 (1.58–5.04)	<0.001	2.79 (1.57–4.96)	<0.001
Q4	2.68 (1.85–3.89)	<0.001	2.45 (1.67–3.59)	<0.001	2.57 (1.19–5.55)	0.016	2.63 (1.22–5.64)	0.013

HR: Hazard Ratio, CI: Confidence Interval.

Model1: Crude.

Model2: Adjust: age, gender, insurance, marital_status, heart_rate, sbp, dbp, spo2.

Model3: Adjust: age, gender, insurance, marital_status, wbc, basophils_abs, eosinophils_abs, lymphocytes_abs, monocytes_abs, neutrophils_abs, basophils, eosinophils, lymphocytes, monocytes, neutrophils, hematocrit, hemoglobin, mch, mchc, mcv, platelet, rbc, rdw, albumin, aniongap, bicarbonate, bun, calcium, chloride, creatinine, glucose, sodium, potassium, crp, alt, alp, ast, amylase, bilirubin_total, bilirubin_direct, bilirubin_indirect, ck_cpk, ck_mb, ld_ldh, lactate, apsiii, heart_rate, sbp, dbp, spo2, urineoutput_24 h, gcs.

Model4: Adjust: age, gender, insurance, marital_status, wbc, basophils_abs, eosinophils_abs, lymphocytes_abs, monocytes_abs, neutrophils_abs, basophils, eosinophils, lymphocytes, monocytes, neutrophils, hematocrit, hemoglobin, mch, mchc, mcv, platelet, rbc, rdw, peripheral_vascular_disease, cerebrovascular_disease, dementia, chronic_pulmonary_disease, rheumatic_disease, peptic_ulcer_disease, mild_liver_disease, diabetes_with_cc, paraplegia, renal_disease, malignant_cancer, severe_liver_disease, metastatic_solid_tumor, aids, albumin, aniongap, bicarbonate, bun, calcium, chloride, creatinine, glucose, sodium, potassium, crp, alt, alp, ast, amylase, bilirubin_total, bilirubin_direct, bilirubin_indirect, ck_cpk, ck_mb, ld_ldh, lactate, apsiii, heart_rate, sbp, dbp, spo2, urineoutput_24 h.

### Restricted cubic spline curve

**FIGURE 2:**
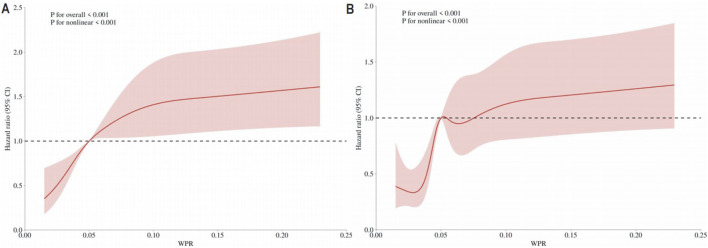
**(A)** RCS Results for 28-Day Mortality The curve represents the estimated adjusted hazard ratios, with the shaded bands indicating the 95% confidence intervals. The vertical dashed line indicates the lowest point of the curve, representing the minimum hazard ratio. The horizontal dashed line represents a hazard ratio of 1.0, HR = Hazard Ratio; CI = Confidence Interval; **(B)** RCS Results for In-Hospital Mortality The curve represents the estimated adjusted hazard ratios, with the shaded bands indicating the 95% confidence intervals. The vertical dashed line indicates the lowest point of the curve, representing the minimum hazard ratio. The horizontal dashed line represents a hazard ratio of 1.0, HR = Hazard Ratio; CI = Confidence Interval.

RCS analysis was adjusted for age, gender, insurance, marital status, wbc, basophils_abs, eosinophils_abs, lymphocytes_abs, monocytes_abs, neutrophils_abs, basophils, eosinophils, lymphocytes, monocytes, neutrophils, hematocrit, hemoglobin, mch, mchc, mcv, platelet, rbc, rdw, peripheral_vascular_disease, cerebrovascular_disease, dementia, chronic_pulmonary_disease, rheumatic_disease, peptic_ulcer_disease, mild_liver_disease, diabetes_with_cc, paraplegia, renal_disease, malignant_cancer, severe_liver_disease, metastatic_solid_tumor, aids, albumin, aniongap, bicarbonate, bun, calcium, chloride, creatinine, glucose, sodium, potassium, crp, alt, alp, ast, amylase, bilirubin_total, bilirubin_direct, bilirubin_indirect, ck_cpk, ck_mb, ld_ldh, lactate, apsiii, heart_rate, sbp, dbp, and spo2. RCS analysis for 28-day all-cause mortality (Figure 2A) and in-hospital mortality (Figure 2B) both indicated a U-shaped association between WPR and mortality risk.

#### KM survival analysis

**FIGURE 3:**
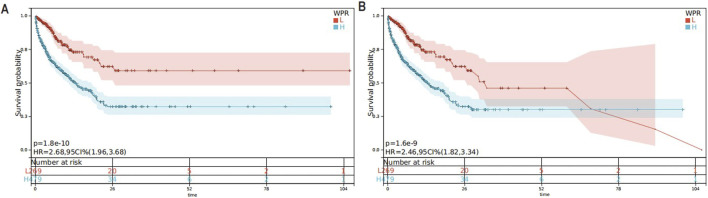
**(A)** KM Survival Curve for 28-Day In-Hospital Mortality,The Kaplan-Meier curve displays the survival rates within 28 days for patients with high and low levels of WPR; **(B)** KM Survival Curve for In-Hospital Mortality, The Kaplan-Meier curve displays the survival rates within 28 days for patients with high and low levels of WPR.

In this study, we utilized Kaplan-Meier survival curves to analyze the impact of different WPR levels in CA patients. By distinguishing between high and low WPR groups based on the median WPR, we compared the survival probabilities of patients with high (H group) and low (L group) WPR levels. We found that the in-hospital mortality and 28-day in-hospital mortality were significantly lower in the high WPR group compared to the low WPR group throughout the observation period (*p* = 1.6e-9 and *p* = 1.8e-10, respectively). Specifically, the hazard ratios for the high WPR group were 2.68 and 2.46 (Figures 3A, B), indicating that patients with high WPR levels had a significantly higher risk of death compared to those with low WPR levels.

### Association between WPR and 28-day mortality

In this study, the relationship between WPR quartiles and survival rates in CA patients was analyzed through four models with progressively adjusted variables. The first model (Model 1) was unadjusted and showed that the highest quartile (Q4) had the highest risk of death (HR = 2.68, *p* < 0.001) compared to the reference group (Q1). As the models progressed (from Model 2 to Model 4), additional adjustments were made for age, gender, insurance, marital status, and various physiological and biochemical indicators. Although the hazard ratios slightly decreased, they remained statistically significant, indicating a persistent negative association between WPR and patient survival across diverse clinical and biochemical contexts. Model 4, the most comprehensive model, included a wide range of clinical and biochemical indicators as well as various disease states as adjusting variables. This model continued to show that patients in the Q4 group had a significantly higher risk of death compared to the reference group (HR = 2.63, *p* = 0.013). These results underscore the potential of high WPR as an independent predictor of poor prognosis in CA patients, with its impact remaining significant even after adjusting for multiple factors.

### Association between WPR and in-hospital mortality

This study explored the relationship between WPR and 28-day all-cause mortality in CA patients. The results indicated that patients with higher WPR had a significantly increased 28-day all-cause mortality rate (Table 6). In the unadjusted Model 1, the hazard ratios (HR) for the WPR quartiles ranged from 1.88 (95% CI: 1.22–2.90) in Q2 to 3.02 (95% CI: 2.04–4.47) in Q4 (Ptrend <0.05 for all). After adjusting for age, gender, insurance status, marital status, heart rate, systolic blood pressure, diastolic blood pressure, and blood oxygen saturation in Model 2, the adjusted HRs were 1.78 (95% CI: 1.15–2.77) for Q2 and 2.70 (95% CI: 1.81–4.03) for Q4 (Ptrend <0.05 for all).Further adjustments for various biochemical indicators and clinical parameters in Models 3 and 4 yielded consistent results, with the highest WPR group (Q4) having HRs of 3.08 (95% CI: 1.39–6.84) and 3.15 (95% CI: 1.43–6.95), respectively (Ptrend <0.05 for all). In summary, a higher WPR is significantly associated with increased 28-day all-cause mortality in CA patients, suggesting that WPR could be an important prognostic indicator for outcome assessment.

**TABLE 6:** Cox regression model (in-hospital mortality).

Variables	Model1		Model2		Model3		Model4	
	HR (95%CI)	Ptrend	HR (95%CI)	Ptrend	HR (95%CI)	Ptrend	HR (95%CI)	Ptrend
WPR quantile								
Q1	1.00 (Reference)		1.00 (Reference)		1.00 (Reference)		1.00 (Reference)	
Q2	1.88 (1.22–2.90)	0.004	1.78 (1.15–2.77)	0.01	2.09 (1.22–3.57)	0.007	2.09 (1.22–3.57)	0.007
Q3	2.58 (1.73–3.84)	<0.001	2.26 (1.49–3.43)	<0.001	3.26 (1.79–5.94)	<0.001	3.22 (1.77–5.85)	<0.001
Q4	3.02 (2.04–4.47)	<0.001	2.70 (1.81–4.03)	<0.001	3.08 (1.39–6.84)	0.006	3.15 (1.43–6.95)	0.004

HR: Hazard Ratio, CI: Confidence Interval.

Model1: Crude.

Model2: Adjust: age, gender, insurance, marital_status, heart_rate, sbp, dbp, spo2.

Model3: Adjust: age, gender, insurance, marital_status, wbc, basophils_abs, eosinophils_abs, lymphocytes_abs, monocytes_abs, neutrophils_abs, basophils, eosinophils, lymphocytes, monocytes, neutrophils, hematocrit, hemoglobin, mch, mchc, mcv, platelet, rbc, rdw, albumin, aniongap, bicarbonate, bun, calcium, chloride, creatinine, glucose, sodium, potassium, crp, alt, alp, ast, amylase, bilirubin_total, bilirubin_direct, bilirubin_indirect, ck_cpk, ck_mb, ld_ldh, lactate, apsiii, heart_rate, sbp, dbp, spo2, urineoutput_24h, gcs.

Model4: Adjust: age, gender, insurance, marital_status, wbc, basophils_abs, eosinophils_abs, lymphocytes_abs, monocytes_abs, neutrophils_abs, basophils, eosinophils, lymphocytes, monocytes, neutrophils, hematocrit, hemoglobin, mch, mchc, mcv, platelet, rbc, rdw, peripheral_vascular_disease, cerebrovascular_disease, dementia, chronic_pulmonary_disease, rheumatic_disease, peptic_ulcer_disease, mild_liver_disease, diabetes_with_cc, paraplegia, renal_disease, malignant_cancer, severe_liver_disease, metastatic_solid_tumor, aids, albumin, aniongap, bicarbonate, bun, calcium, chloride, creatinine, glucose, sodium, potassium, crp, alt, alp, ast, amylase, bilirubin_total, bilirubin_direct, bilirubin_indirect, ck_cpk, ck_mb, ld_ldh, lactate, apsiii, heart_rate, sbp, dbp, spo2, urineoutput_24h.

### Subgroup and sensitivity analysis

The results of the subgroup analysis for 28-day all-cause mortality are presented in Table 7. In this study, we conducted a detailed subgroup analysis to investigate the impact of the WPR on the survival rate of CA patients, considering factors such as gender, age, insurance type, marital status, and various health conditions.The findings revealed that a high WPR was significantly associated with an increased risk of death in male patients (HR = 2.26, *p* = 0.005), while this association was not significant in female patients (HR = 1.5, *p* = 0.232). For age, the ≥65 years old group showed a marginally significant high risk (*p* = 0.01), but there was no significant impact in younger patients (HR = 1.73, *p* = 0.128). Among insurance types, Medicaid beneficiaries exhibited a higher hazard ratio, although it lacked statistical significance (HR = 6.46, *p* = 0.107). In married patients, the risk associated with WPR was significantly increased (HR = 2.45, *p* = 0.007), whereas no significant association was observed in other marital statuses. In patients with chronic lung disease, a high WPR was significantly associated with a higher risk of death (HR = 2.48, *p* = 0.001). These results indicate that the impact of WPR is influenced by multiple factors, including gender, age, marital status, and health conditions. Specifically, high WPR is related to significantly increased mortality risk in male, married, elderly patients, and those with chronic lung disease. This finding underscores the importance of considering a wide range of patient backgrounds and health statuses in the clinical management of CA patients, to more accurately assess risk and formulate appropriate treatment strategies.

**TABLE 7:** Subgroup analysis of 28-day mortality.

Variable	Count	Percent	HR	Lower	Upper	Q1	Q2	Q3	Q4	*p*-Value	P For interaction
Overall	748	100	1.88	1.22	2.9	40	68.4	72.8	60.3	004	
gender											0.528
Male	459	61.4	2.26	1.29	3.99	34.9	73.8	78	57.7	005	
Female	289	38.6	1.5	0.77	2.94	47.8	60.4	68.7	64.5	0.232	
age											0.841
≥65	420	56.1	25	1.19	3.54	46.6	84.5	82.6	68	01	
<65	328	43.9	1.73	0.85	3.51	31.7	50.9	56.6	52.1	0.128	
insurance											0.512
Medicaid	42	5.6	6.46	0.67	62.33	12.5	65.7	81.2	40	0.107	
Medicare	382	51.1	1.7	0.93	3.11	51.6	65.7	69.2	60.2	083	
Other	324	43.3	1.85	0.97	3.53	35.8	74.5	72.8	65.8	06	
marital_status											0.326
DIVORCED	45	6	1.59	0.14	17.6	33.3	33.3	38.5	85	0.705	
MARRIED	373	49.9	2.45	1.28	4.67	35.2	74.3	72.1	60.6	007	
SINGLE	246	32.9	1.77	0.86	3.64	40.4	61.6	74.5	49.1	0.123	
WIDOWED	84	11.2	0.86	0.29	2.57	70.3	100	84	76.5	0.792	
myocardial_infarct											0.561
No	521	69.7	1.6	0.98	2.6	49.8	66.6	71.1	60.8	061	
Yes	227	30.3	3.36	1.3	8.7	19.4	84.1	76.5	59.9	013	
congestive_heart_failure											0.29
No	381	50.9	1.92	1.1	3.35	53.2	75.5	78.9	72.4	022	
Yes	367	49.1	1.79	0.9	3.54	24.3	61	65	41.6	095	
peripheral_vascular_disease											066
No	636	85	1.55	0.98	2.46	42.8	67.3	72.5	58.9	062	
Yes	112	15	11.13	1.45	85.11	20	72.8	73.8	70.7	02	
cerebrovascular_disease											019
No	642	85.8	1.48	0.94	2.34	43.8	64.8	73.8	59.1	091	
Yes	106	14.2	15.64	21	121.4	20	84.4	68.8	67.7	009	
dementia											0.898
No	718	96	1.86	1.2	2.89	40.6	68.6	70.3	59.9	006	
Yes	30	4	2.44	0.25	23.65	33.3	65.7	100	72.2	0.441	
chronic_pulmory_disease											069
No	561	75	2.48	1.45	4.23	38.7	70.8	74.6	65.3	001	
Yes	187	25	11	0.46	2.23	40.5	63.1	69.7	47.6	0.976	
rheumatic_disease											068
No	717	95.9	1.9	1.22	2.94	39.3	69.9	72	60.4	004	
Yes	31	4.1	1.92	0.17	21.33	100	33.3	85.7	60	0.595	
peptic_ulcer_disease											0.807
No	728	97.3	1.89	1.22	2.94	40.5	68.4	72.7	61	004	
Yes	20	2.7	1.71	0.14	20.83	37.5	25	50	50	0.674	
mild_liver_disease											0.49
No	645	86.2	1.95	1.22	3.11	38.8	72.3	74.5	58	005	
Yes	103	13.8	1.52	0.49	4.72	44	45.2	68.9	75.4	0.468	
diabetes_without_cc											0.179
No	557	74.5	1.44	0.88	2.36	51.2	66.3	77.2	59.8	0.147	
Yes	191	25.5	3.72	1.46	9.47	15	77.5	56.5	60.7	006	
diabetes_with_cc											0.732
No	625	83.6	1.77	1.1	2.85	45.7	67.9	74.9	63	018	
Yes	123	16.4	2.6	0.91	7.42	19.6	74.1	40.2	33.1	074	
paraplegia											0.167
No	715	95.6	1.88	1.21	2.92	40.8	68.2	69	62.4	005	
Yes	33	4.4	2.12	0.29	15.41	42.9	60	87.5	31.4	0.458	
rel_disease											0.326
No	493	65.9	1.59	0.94	2.7	46.9	69.4	69.4	62.4	082	
Yes	255	34.1	2.61	1.22	5.59	31.6	67.9	80.3	53.7	013	
malignt_cancer											0.759
No	691	92.4	1.84	1.16	2.9	43.5	71.6	72.5	58.8	009	
Yes	57	7.6	29	0.59	7.43	26.7	48.6	81	75.7	0.254	
severe_liver_disease											0.417
No	725	96.9	1.83	1.18	2.85	39.8	67.7	70.2	58.7	007	
Yes	23	3.1	3.47	0.38	31.35	33.3	79.2	100	100	0.268	
metastatic_solid_tumor											0.267
No	729	97.5	23	1.29	3.19	34.7	68	72.6	59.6	002	
Yes	19	2.5	0.66	0.14	3.11	100	75	66.7	62.5	0.602	

#### Adjust

wbc, basophils_abs, eosinophils_abs, lymphocytes_abs, monocytes_abs, neutrophils_abs, basophils, eosinophils, lymphocytes, monocytes, neutrophils, hematocrit, hemoglobin, mch, mchc, mcv, platelet, rbc, rdw, paraplegia, renal_disease, malignant_cancer, severe_liver_disease, metastatic_solid_tumor, aids, albumin, aniongap, bicarbonate, bun, calcium, chloride, creatinine, glucose, sodium, potassium, crp, alt, alp, ast, amylase, bilirubin_total, bilirubin_direct, bilirubin_indirect, ck_cpk, ck_mb, ld_ldh, lactate, apsiii, heart_rate, sbp, dbp, spo2, urineoutput_24 hr.

## Discussion

CA is one of the most critical conditions in clinical practice, characterized by extremely high mortality rates. Even with timely and effective cardiopulmonary resuscitation (CPR), survival rates remain low. In recent years, numerous studies have demonstrated that hematological and biochemical indicators can effectively predict the prognosis of CA.

The WPR has garnered attention as a new inflammatory marker. This study used a retrospective cohort analysis to investigate the relationship between WPR and 28-day all-cause mortality in CA patients, and to evaluate the impact of various hematological indicators on the prognosis of CA.

The WPR has been increasingly recognized as a valuable prognostic marker in critically ill patients, including those who have experienced CA. WPR combines two key components of the immune and coagulation systems—white blood cells (which mediate inflammation) and platelets (which contribute to clotting and inflammation). These two systems play crucial roles in determining patient outcomes after severe events like CA, where systemic inflammation and hemostatic imbalance are common. In the context of CA, WPR is particularly relevant because elevated levels of both white blood cells and platelets are often associated with adverse outcomes, including poor recovery and increased mortality. Inflammatory markers, such as white blood cell count, reflect the body’s immune response to stressors like tissue injury and ischemia, which are common in CA. Similarly, platelets, beyond their role in coagulation, contribute to inflammation by releasing cytokines and interacting with immune cells. High WPR, therefore, serves as an indicator of an exaggerated inflammatory and coagulative response, both of which are implicated in multi-organ failure and poor survival following CA.

Our study demonstrates that higher WPR is significantly associated with 28-day all-cause mortality in CA patients. This finding aligns with previous research, which has shown that elevated WPR predicts poor outcomes in other critical conditions. By measuring WPR, clinicians can identify patients at higher risk of mortality and tailor their treatment strategies accordingly. Elevated WPR may signal the need for more aggressive or targeted interventions aimed at managing inflammation and preventing further deterioration in these high-risk patients.

We employed a series of methods for data analysis and feature selection. Initially, detailed patient information was extracted using pgAdmin software, including age, gender, insurance, marital status, length of hospital stay, white blood cell count, platelet count, and various biochemical and hematological indicators. The Cox proportional hazards model was used to evaluate the impact of each feature on survival time.

In the feature selection stage, we used the Boruta (Adrie et al., 2002) algorithm and random forest algorithm (Erol et al., 2019), both machine learning-based methods, to effectively identify features most relevant to prognosis. We analyzed data from 500 random forest trees, identifying WPR, mean corpuscular hemoglobin concentration (MCHC), red cell distribution width coefficient of variation (RDW-CV), and lymphocyte to monocyte ratio (LMR) as key features. To further understand the impact mechanisms of these features, we introduced SHAP value analysis. SHAP values can explain the contribution of each feature to the model’s prediction results. By calculating SHAP values using the treeshap package, we visualized the importance and influence paths of each feature in the model. WPR consistently ranked highest in importance across Boruta, RF, and SHAP analyses.

For further analysis, we examined data from 748 CA patients, divided into four groups based on WPR quartiles. The study found that age (mean 65.88 years ±16.44, F = 1.69, *p* = 0.167) and length of hospital stay (8.22–10.22 days, F = 1.53, *p* = 0.204) did not show significant statistical differences. However, white blood cell count was significantly higher in the fourth group (F = 161.27, *p* < 0.001), suggesting a stronger inflammatory response in the high WPR group. Other significantly different indicators included absolute eosinophil count, absolute lymphocyte count, and absolute and relative neutrophil counts (all *p* < 0.001). This may be due to the cessation of forward arterial blood flow during CA, leading to zero oxygen flow to tissues, causing systemic ischemia and metabolic failure. CPR can only partially counteract this, achieving 25% of pre-arrest cardiac output. In patients achieving return of spontaneous circulation (ROSC), reperfusion causes a surge in reactive oxygen species (ROS) and sterile inflammation (Adrie et al., 2002). Sterile inflammation recruits pro-inflammatory immune cells, with ischemic tissues releasing damage-associated molecular patterns recognized by the innate immune system, triggering the post-CA inflammatory cascade. Other triggers include lipopolysaccharides (LPS) translocating into the bloodstream due to ischemic gut mucosa, leading to increased white blood cells, which are closely associated with adverse reactions and poor prognosis in CA (Cunningham et al., 2022).

Moreover, for hematological parameters, hematocrit did not show significant differences (F = 1.11, *p* = 0.346), but hemoglobin levels approached significance (F = 2.57, *p* = 0.054). MCH and MCHC exhibited significant differences (MCH F = 13.64, *p* < 0.001; MCHC F = 9.43, *p* < 0.001), suggesting these indicators have potential value in assessing the prognosis of CA. Platelet count showed significant variations among groups, with Group 1 having the highest count (F = 45.87, *p* < 0.001). MCHC reflects the average concentration of hemoglobin in a given volume of red blood cells, currently considered a reliable indicator of iron load in red blood cells. Low MCHC is most commonly caused by anemia or iron deficiency, but kidney function may influence MCHC measurements through volume control. Theoretically, osmotic changes in congestive conditions might affect the relative concentration of hemoglobin within red blood cells, explaining the observation of low MCHC in later-stage renal insufficiency, making patients more prone to fluid retention. Changes in hemoglobin and red blood cell parameters might be related to insufficient tissue oxygen supply, as CA-induced myocardial arrest reduces oxygen delivery. Furthermore, changes in MCH and MCHC might reflect adjustments in hemoglobin content and concentration within red blood cells, possibly related to stress response and metabolic regulation in the body. Significant differences in platelet count suggest differences in coagulation function and stress response among patients, correlating with prognosis and survival rates.

In the Cox proportional hazards model analysis, we found that WPR was significantly associated with 28-day all-cause mortality in CA patients. In the unadjusted Model 1, hazard ratios (HR) for WPR quartiles ranged from 1.88 (95% CI: 1.22–2.90) in Q2 to 3.02 (95% CI: 2.04–4.47) in Q4 (Ptrend <0.05). In Model 2, adjusted for age, gender, insurance status, marital status, heart rate, systolic blood pressure, diastolic blood pressure, and blood oxygen saturation, the HRs were 1.78 (95% CI: 1.15–2.77) for Q2 and 2.70 (95% CI: 1.81–4.03) for Q4 (Ptrend <0.05). Further adjustments in Models 3 and 4 for various biochemical indicators and clinical parameters still yielded significant results, with the highest WPR group (Q4) having HRs of 3.08 (95% CI: 1.39–6.84) and 3.15 (95% CI: 1.43–6.95), respectively (Ptrend <0.05). In summary, higher WPR is significantly associated with 28-day all-cause mortality in CA patients, suggesting that WPR could be an important prognostic indicator.

To further understand the impact of WPR on prognosis, we conducted a restricted cubic spline (RCS) analysis. The RCS analysis showed a U-shaped association between WPR and mortality risk, further validating the relationship between high WPR and poor prognosis. Kaplan-Meier curves also indicated that low WPR was associated with higher survival rates (Zelniker et al., 2021).

We conducted subgroup analyses based on gender, age, insurance type, marital status, and health conditions to explore the impact of WPR on survival rates across different subgroups. The results showed that high WPR was significantly associated with increased mortality risk in male patients (HR = 2.26, *p* = 0.005), while the association was not significant in female patients (HR = 1.5, *p* = 0.232). For age, the ≥65 years group showed a marginally significant high risk (*p* = 0.01), but there was no significant impact in younger patients (HR = 1.73, *p* = 0.128). Among insurance types, Medicaid beneficiaries exhibited a higher hazard ratio, although it lacked statistical significance (HR = 6.46, *p* = 0.107). In married patients, the risk associated with WPR was significantly increased (HR = 2.45, *p* = 0.007), whereas no significant association was observed in other marital statuses. In patients with chronic lung disease, high WPR was significantly associated with a higher risk of death (HR = 2.48, *p* = 0.001).

These results indicate that the impact of WPR is influenced by multiple factors, including gender, age, marital status, and health conditions. Specifically, high WPR is related to significantly increased mortality risk in male, married, elderly patients, and those with chronic lung disease.

The study results suggest that the impact of WPR on survival rates varies significantly across different subgroups. The higher risk for cardiovascular diseases in males might be due to higher smoking rates, unhealthy dietary habits, and lower utilization of healthcare services. Additionally, lower estrogen levels in males might lead to weaker cardiovascular protection. High WPR is associated with increased mortality risk (HR = 1.5), but it is not statistically significant (*p* = 0.232). This could be due to insufficient sample size or the physiological differences in cardiovascular risk in females, such as stronger cardiovascular protection from estrogen (Sandroni et al., 2018; Noppens et al., 2009). High WPR shows a marginally significant increase in mortality risk (*p* = 0.01). Elderly people often have multiple health issues (e.g., chronic diseases, reduced immune function), which may increase inflammatory responses, thus making the association between WPR and mortality more pronounced. Although high WPR also shows a trend towards increased mortality risk (HR = 1.73), it is not statistically significant (*p* = 0.128). The relatively lower baseline health risks in younger individuals might mitigate the impact of WPR on mortality rates. A higher hazard ratio (HR = 6.46) is observed, although it is not statistically significant (*p* = 0.107). This might reflect poorer health status, fewer medical resources, and economic difficulties negatively impacting health outcomes among Medicaid beneficiaries. High WPR significantly increases mortality risk (HR = 2.45, *p* = 0.007). Married status usually provides better social support and mental health, but in cases of high WPR, it might indicate more severe health problems under high stress and disease burden. Other marital statuses do not show significant differences, reflecting the complexity and diversity of health impacts across different marital statuses. High WPR is significantly associated with higher mortality risk (HR = 2.48, *p* = 0.001). Chronic lung disease often accompanies long-term inflammatory responses and immune system activation (Benjamin et al., 2019), which may enhance the association between WPR and mortality.

Our study’s findings, which identify a significant relationship between the white blood cell-to-platelet ratio (WPR) and 28-day all-cause mortality in CA patients, are consistent with several key studies that have explored the prognostic value of WPR in critically ill populations. For instance, a study showed that WPR is associated with poor outcomes, including higher mortality rates, in critically ill patients, making it a reliable marker for predicting adverse outcomes. Similarly, researchers found that elevated WPR was significantly linked to poor prognosis in patients with acute coronary syndrome, reinforcing the utility of WPR across different cardiovascular conditions (Zhang et al., 2019). Furthermore, A study emphasized the value of WPR in predicting poor outcomes in sepsis and septic shock patients, which share common inflammatory pathways with CA (Schupp et al., 2023). These studies collectively underscore the potential of WPR as a robust, easily accessible biomarker for predicting mortality in various critical conditions, including CA.

Although this study reveals the important role of WPR in the prognosis of CA, there are still some limitations. First, this is a single-center retrospective study, which might introduce selection bias. Second, although we adjusted for multiple potential confounders, other unknown factors could not be entirely ruled out. Therefore, more multicenter, large-sample prospective studies are needed in the future to further validate our findings.

## Conclusion

In conclusion, this study found that WPR is significantly associated with 28-day all-cause mortality in CA patients, suggesting that WPR could be an important prognostic indicator. Future research should further explore the value of WPR in different patient groups to provide more evidence for clinical decision-making.

## Data Availability

The original contributions presented in the study are included in the article/supplementary material, further inquiries can be directed to the corresponding authors.

## Abbreviations

CA, Cardiac Arrest; CPR, Cardiopulmonary Resuscitation; WPR, White Blood Cell to Platelet Ratio; MIMIC-IV, Medical Information Mart for Intensive Care-IV; pgAdmin, PostgreSQL Administration Tool; RCS, Restricted Cubic Spline; HR, Hazard Ratio; CI, Confidence Interval; ICU, Intensive Care Unit; ACLS, Advanced Cardiovascular Life Support; NLR, Neutrophil-to-Lymphocyte Ratio; LMR, Lymphocyte-to-Monocyte Ratio; RDW-CV, Red Cell Distribution Width-Coefficient of Variation; MCHC, Mean Corpuscular Hemoglobin Concentration; SHAP, Shapley Additive Explanations; RF, Random Forest; ALT, Alanine Aminotransferase; AST, Aspartate Aminotransferase; CRP, C-Reactive Protein; APSIII, Acute Physiology Score III.
